# Supplementary material for: Evaluating the User Experience and Usability of the MINI Robot for Elderly Adults with Mild Dementia and Mild Cognitive Impairment: Insights and Recommendations
Source: Sensors (Basel). 2024 Nov 8;24(22):7180. doi: 10.3390/s24227180 (PMC11597995; doi:10.3390/s24227180)
Supplement: Supplementary file 1 [file sensors-24-07180-s001.zip › Supplementary Material S1.pdf]

Supplementary Material S1.

System Usability Scale

© Digital Equipment Corporation, 1986.

| | Strongly disagree | | | | Strongly agree |
|---|---|---|---|---|---|
| 1. I think that I would like to use this system frequently | 1 | 2 | 3 | 4 | 5 |
| 2. I found the system unnecessarily complex | 1 | 2 | 3 | 4 | 5 |
| 3. I thought the system was easy to use | 1 | 2 | 3 | 4 | 5 |
| 4. I think that I would need the support of a technical person to be able to use this system | 1 | 2 | 3 | 4 | 5 |
| 5. I found the various functions in this system were well integrated | 1 | 2 | 3 | 4 | 5 |
| 6. I thought there was too much inconsistency in this system | 1 | 2 | 3 | 4 | 5 |
| 7. I would imagine that most people would learn to use this system very quickly | 1 | 2 | 3 | 4 | 5 |
| 8. I found the system very cumbersome to use | 1 | 2 | 3 | 4 | 5 |
| 9. I felt very confident using the system | 1 | 2 | 3 | 4 | 5 |
| 10. I needed to learn a lot of things before I could get going with this system | 1 | 2 | 3 | 4 | 5 |
