# Supplementary material for: Evaluating the User Experience and Usability of the MINI Robot for Elderly Adults with Mild Dementia and Mild Cognitive Impairment: Insights and Recommendations
Source: Sensors (Basel). 2024 Nov 8;24(22):7180. doi: 10.3390/s24227180 (PMC11597995; doi:10.3390/s24227180)
Supplement: Supplementary file 1 [file sensors-24-07180-s001.zip › Supplementary Material S2.pdf]

Supplementary Material S2. SUS results item by item

Item	N	Strongly disagree	Disagree	Neutral	Agree	Strongly agree	Mean	SD
I think I would like to use the robot frequently.	10	0.0%	20.0%	0.0%	50.0%	30.0%	3.90	1.10
I did not find the robot unnecessarily complex.	10	40.0%	40.0%	0.0%	10.0%	10.0%	3.90	1.60
I thought the robot was easy to use.	10	0.0%	30.0%	10.0%	40.0%	20.0%	3.50	1.18
I think that I would not need assistance to be able to use robot.	10	20.0%	20.0%	20.0%	20.0%	20.0%	3.00	1.50
I found the various functions in the product were well integrated.	10	0.0%	0.0%	20.0%	70.0%	10.0%	3.90	.57
I thought there was too much inconsistency in this robot.	10	20.0%	30.0%	30.0%	20.0%	0.0%	3.50	1.43
I imagine that most people learn to use the robot very quickly.	10	0.0%	0.0%	0.0%	60.0%	40.0%	4.40	.52
I did not find the robot very awkward to use.	10	60.0%	30.0%	0.0%	10.0%	0.0%	4.40	.70
I felt very confident using the robot.	10	0.0%	10.0%	40.0%	30.0%	20.0%	3.60	.96
I do not need to learn a lot of things before I use the robot.	10	20.0%	30.0%	10.0%	30.0%	10.0%	3.20	.78
N: number of respondents; SD: standard deviation								
